# Orientation-Dependent Protection by LiF Interlayers at LATP Solid–Electrolyte Interfaces: A First-Principles Study

**DOI:** 10.1021/acsami.6c02962

**Published:** 2026-06-21

**Authors:** Maryam Kookhaee, Ali Lashani Zand, Maryam Soleimani, Nicola Seriani, Shamsoddin Mohajerzadeh, Mahdi Pourfath

**Affiliations:** † School of Electrical and Computer Engineering, College of Engineering, 48425 University of Tehran, Tehran 14395-515, Iran; ‡ Condensed Matter and Statistical Physics Section, The Abdus Salam International Centre for Theoretical Physics, Strada Costiera 11, Trieste 34151, Italy; § Institute for Microelectronics, TU Wien, Gusshausstrasse 27-39/E360, Vienna 1040, Austria

**Keywords:** solid-state lithium metal batteries, LATP solid electrolyte, LiF protective interlayer, electrode−electrolyte interfaces, interfacial transport, first-principles calculations

## Abstract

Solid-state lithium metal batteries require chemically stable and electronically insulating interfaces to suppress interfacial reduction reactions and enable long-term cycling stability. Li_1.3_Al_0.3_Ti_1.7_(PO_4_)_3_ (LATP) exhibits high ionic conductivity but undergoes spontaneous Ti^4+^ reduction when placed in direct contact with a Li metal anode. Here, density functional theory and *ab initio* molecular dynamics are combined with a descriptor-based, physics-informed first-principles analysis to elucidate how crystallographic orientation governs the protective function of LiF coatings on LATP(012) surfaces. Three LiF orientations—(100), (110), and (111)—are systematically compared using formation energetics, layer-resolved projected density of states, Bader charge analysis, charge-density differences, electrostatic potential profiles, interface dipole moments, and interfacial electric-field distributions. Physically motivated descriptors extracted from first-principles outputs enable efficient, data-driven comparison of interfacial polarization and electronic blocking behavior across orientations. Among the orientations considered here, the LiF(100)/LATP(012) interface shows a favorable electronic-blocking response, with no pronounced interface-induced localized electronic states, reduced area-normalized charge redistribution, weaker interfacial polarization, and ordered Li coordination with comparatively low Li-ion diffusivity. In contrast, LiF(110) exhibits pronounced interfacial polarization, formation of interface-induced localized electronic states, enhanced Li–O coordination, and the highest Li-ion diffusivity, indicating stronger electronic and ionic coupling across the interface. *Ab initio* molecular dynamics simulations indicate structural stability within the simulated time scale at room temperature. These results demonstrate that crystallographic orientation is an important factor influencing the electronic and ionic behavior of ultrathin LiF-based protective interlayers on LATP surfaces.

## Graphical abstract

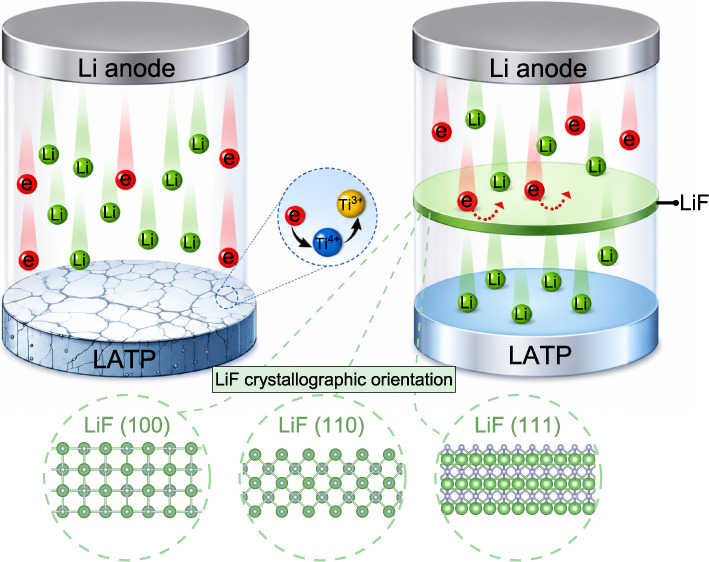

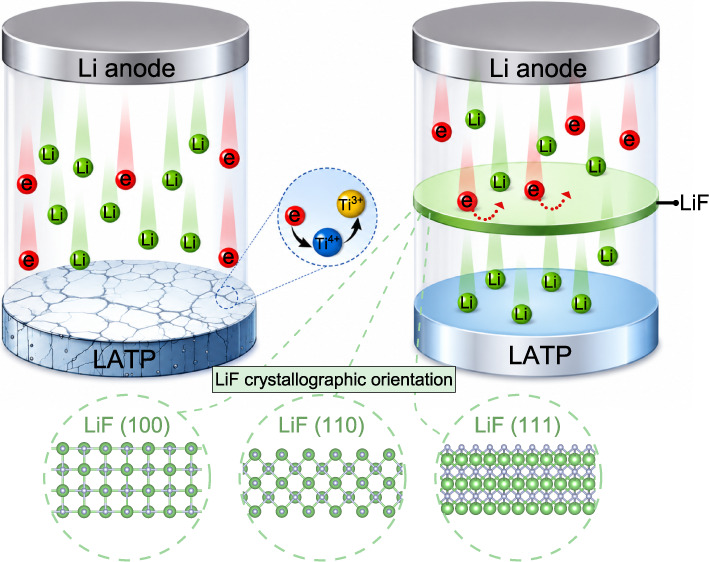

## 1 Introduction

All-solid-state lithium metal batteries (ASSLMBs) are widely regarded as next-generation energy-storage systems owing to their high energy density, intrinsic safety, and improved cycling stability compared with conventional liquid electrolytes.^1,2^ The use of Li metal as an anode further boosts the energy density because of its ultrahigh specific capacity and low redox potential.^3–5^ However, the practical deployment of ASSLMBs is still constrained by severe interfacial challenges, in particular the high interfacial resistance and chemical/electrochemical instability at solid electrolyte (SE)–Li metal and SE–cathode interfaces, which can trigger parasitic reactions, mixed conduction, and mechanical failure.^6–8^

Among oxide-based SEs, NASICON-type Li_1.3_Al_0.3_Ti_1.7_(PO_4_)_3_ (LATP) has attracted considerable attention due to its high room-temperature ionic conductivity (∼10^–3^ S cm^–1^), good air stability, and relatively low activation energy for Li-ion transport.^9–12^ Despite these advantages, LATP exhibits poor chemical and electrochemical compatibility with Li metal anodes. Direct contact between LATP and Li drives spontaneous electron transfer from Li to the Ti-centered framework, reducing Ti^4+^ to Ti^3+^ and forming mixed conducting interphases (MCIs) that increase interfacial resistance and accelerate capacity fade.^13–15^ Suppressing this undesired Ti reduction while maintaining efficient Li-ion transport across the interface is a key prerequisite for implementing LATP in ASSLMBs.^16,17^

To address these interfacial limitations, a wide range of interfacial-engineering strategies have been explored, including thin oxide coatings, atomic layer-deposited films, and halide-based interlayers.^16,18,19^ In particular, Li halide-rich interphases such as LiCl- and LiF-containing layers have emerged as promising protective layers because they combine wide band gaps and chemical robustness while supporting Li-ion transport through defects, grain boundaries, or interfacial pathways.^17,20–25^ Experimental studies have demonstrated that LiF-containing interphases can substantially improve the cycling stability of LATP- and LAGP-based cells by mitigating interfacial decomposition and dendrite formation.^13,20,24,25^ In parallel, density functional theory (DFT) investigations have clarified how band alignment, space-charge formation, and defect chemistry govern electron- and ion-transfer across LATP/electrodes and halide/LATP interfaces.^17–19,22^ These works provide important design rules for stabilizing LATP, but they typically consider amorphous or idealized halide layers and do not systematically address how the crystallographic orientation of LiF influences interfacial electronic structure, adhesion, and Li-ion transport.

In realistic devices, LiF often crystallizes with preferred orientations determined by the underlying substrate and growth conditions, and several studies have reported orientation-dependent properties of LiF in electrolytes and on electrode surfaces.^23,26–28^ Because LiF is a wide-band gap ionic crystal, its surface atomic configuration and polarity can strongly affect local band bending, charge transfer, and Li coordination at buried interfaces. For LATP-based cells, these effects are particularly critical: an optimal LiF orientation should suppress parasitic electronic conduction and Ti reduction while still supporting Li-ion transport across the interface. Yet, to the best of our knowledge, a systematic, atomistically resolved comparison of different LiF orientations on LATP surfaces has not been reported, and the role of LiF crystallography in controlling interfacial stability and transport remains largely unexplored.

Here, we address this gap by combining DFT and ab initio molecular dynamics (AIMD) to investigate orientation-dependent protection by LiF interlayers on LATP(012) surfaces. We construct explicit LiF/LATP heterostructures with LiF(100), LiF(110), and LiF(111) overlayers on LATP(012) and perform a comprehensive analysis of their structural, electronic, and ionic properties. Formation energetics and adhesion energies quantify the mechanical compatibility of each interface. Layer-resolved projected density of states (PDOS), band alignment, Bader charge analysis, charge density difference (CDD), and electron localization function (ELF) mapping reveal how a crystallographic orientation governs electronic insulation, charge transfer, and interfacial bonding. AIMD simulations at 300 K, combined with mean-square displacement (MSD) and radial distribution function (RDF) analyses, provide Li-ion diffusion coefficients and local coordination environments at the interfaces. We show that, among the ultrathin LiF/LATP(012) interfaces considered here, LiF(100)/LATP(012) offers a favorable combination of a wide interfacial band gap, reduced charge redistribution, absence of pronounced interface-induced localized electronic states, and ordered Li coordination with lower Li diffusion, whereas LiF(110) and LiF(111) exhibit stronger electronic hybridization, enhanced Li–O interactions, and higher Li mobility. These results demonstrate that the crystallographic orientation is an important factor governing the electronic and ionic behaviors of LiF-based protective interlayers on LATP surfaces and provide atomistic insights into interfacial stabilization in solid-state lithium-metal batteries.

## 2 Computational Methods

All first-principles calculations were performed within DFT as implemented in the Vienna *Ab initio* Simulation Package (VASP) using the projector augmented-wave (PAW) method.^29,30^ Exchange–correlation effects were described using the generalized gradient approximation (GGA) with the Perdew–Burke–Ernzerhof (PBE) functional.^31^ Long-range dispersion interactions in the heterostructure models were included using the DFT-D3 method with Becke–Johnson damping.^17,32,33^ A plane-wave kinetic-energy cutoff of 500 eV was employed throughout all calculations.

Brillouin-zone sampling was performed using a 6 × 6 × 2 Monkhorst–Pack *k*-point mesh for bulk LATP and a 3 × 1 × 1 mesh for the LiF/LATP interface supercells. Structural relaxations were continued until the total-energy change was below 10^–5^ eV and the residual atomic forces were smaller than 0.05 eV Å^–1^.^34–37^

The LiF/LATP interface models were constructed using asymmetric slab geometries separated by a vacuum region of approximately 20 Å along the surface-normal direction to suppress spurious interactions between periodic images. The LiF(100)/LATP(012), LiF(110)/LATP(012), and LiF(111)/LATP(012) heterostructures contain 412, 420, and 460 atoms, respectively, with the corresponding supercell dimensions of approximately 25.08 × 8.37 × 42.72 Å^3^, 26.85 × 8.45 × 42.01 Å^3^, and 28.04 × 8.90 × 44.27 Å^3^. For each interface model, the slab terminations were carefully designed to preserve overall stoichiometry and charge neutrality, thereby avoiding artificial uncompensated surface charges and spurious electrostatic fields associated with polar discontinuities. Dipole corrections were applied along the slab-normal direction to compensate for the artificial electric field arising from the net dipole moment of the asymmetric slab under periodic boundary conditions.^38–40^ Planar-averaged electrostatic potentials were used to identify the macroscopic vacuum plateau within the vacuum region of the slab supercells. This plateau served as a common reference level for evaluating electrostatic potential offsets and orientation-dependent band alignment across the LiF/LATP heterostructures.^38,39,41^

Layer-resolved PDOS was obtained from the self-consistent electronic structure of each relaxed interface supercell and plotted using an energy scale referenced to the Fermi level of the corresponding heterostructure. Because all projected spectra within each interface originate from the same self-consistent calculation, they share a common internal reference energy.

Lithium-vacancy formation energies were evaluated according to

1 $$E_{v}=E_{d}-E_{p}+\mu_{Li}$$

where *E*_d_ and *E*_p_ denote the total energies of the defective and pristine structures, respectively, and μ_Li_ represents the chemical potential of lithium referenced to bulk Li metal.

AIMD simulations were performed in the canonical (*NVT*) ensemble at 300 K using a Langevin thermostat with a friction coefficient of 1 ps^–1^ and a time step of 1 fs for a total simulation time of 20 ps.^26–28,42,43^ The first 5 ps of each AIMD trajectory were discarded as equilibration.^44^ Details of the electrostatic descriptor framework used to quantify charge redistribution, potential alignment, dipole formation, and electric-field response across the LiF/LATP interfaces are summarized in Table S1 of the Supporting Information. One representative LATP-containing interface model was additionally examined using DFT + *U* within the rotationally invariant Dudarev approach, with the Hubbard correction applied to Ti 3d states (see the Supporting Information).

## 3 Results and Discussion

### 3.1 Bulk Electronic Structure and Vacancy Energetics

Before analyzing the interfacial properties of the LiF/LATP heterostructures, PDOS and band gap calculations were performed for the individual bulk structures to establish their intrinsic electronic characteristics.^17^ Both LiF and LATP were fully optimized by using DFT to obtain accurate lattice parameters prior to further analysis.

LiF adopts a rock-salt structure with space group *Fm*3̅*m* and an optimized lattice constant of *a* = *b* = *c* ≈ 4.08 Å, in excellent agreement with previous reports.^45^ Each Li^+^ ion is octahedrally coordinated by six F^–^ ions, with uniform Li–F bond lengths of ∼2.04 Å. This ordered configuration and wide band gap (∼8.7 eV) render LiF chemically stable and electronically insulating.^45^

As shown in Figure 1a, LATP crystallizes in a rhombohedral NASICON-type structure with space group *R*3̅*c* and optimized lattice parameters of *a* = *b* = 8.62 Å and *c* = 21.05 Å, consistent with experimental and theoretical values.^46^ Lithium ions primarily occupy 6b sites, while 18e and 36f sites provide interconnected diffusion pathways that enable efficient Li-ion transport (Figure 1b).^37^ In the Al-doped composition (*x* = 0.33), Al atoms substitute Ti at the 12c sites, and two additional Li ions occupy 18e sites to maintain charge neutrality, stabilizing the conduction channels.^38^ This cation distribution preserves structural integrity and enhances ionic conductivity. The optimized structure contains 8 Li, 2 Al, 10 Ti, 18 P, and 72 O atoms, consistent with the stoichiometry of LATP.

**Figure 1:**
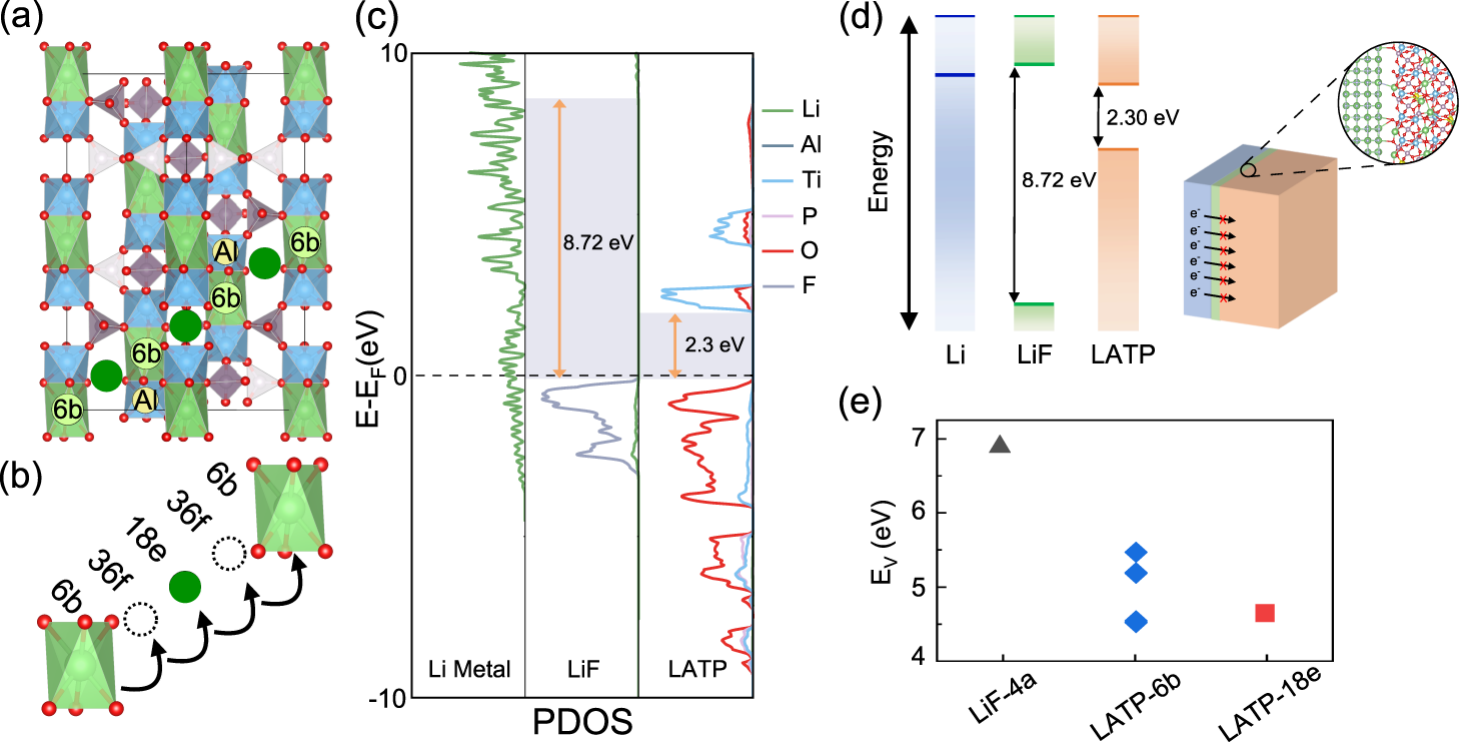
(a) LATP crystal structure with a NASICON symmetry (*R*3̅*c*), where Li occupies the 6b, 18e, and 36f sites forming interconnected diffusion pathways. (b) Li-ion migration channels associated with the 6b and 18e sites. (c) PDOS of LATP, LiF, and Li, showing the LATP band gap, insulating LiF, and metallic Li. (d) Relative band alignment illustrating interfacial driving forces and the electron-blocking role of LiF. (e) Lithium-vacancy formation energies in Al-doped LATP and LiF, showing low *E*_V_ at LATP 18e sites that promote Li transport and higher *E*_V_ in LiF that stabilizes Li while maintaining insulating behavior.

Figure 1c shows the PDOS of Li metal, LiF, and LATP. The latter exhibits a band gap of ∼2.3 eV, with O states dominating the valence band and Ti states the conduction band, confirming its semiconducting nature that suppresses electronic conductivity while supporting Li-ion transport. LiF displays a wide band gap of 8.72 eV, confirming its insulating nature and suitability as an electronic barrier at the LATP/Li interface. By contrast, lithium metal shows metallic behavior, with a continuous density of states across the Fermi level.^17,22^

Figure 1d presents the relative energy alignment of LATP, LiF, and the lithium metal. The offset between Li and LATP reveals a strong driving force for electron migration from Li to LATP, which can trigger interfacial instability in the absence of protection.^14^ Serving as a wide-gap insulator, LiF blocks direct electronic interactions, mitigates interfacial degradation, and suppresses charge accumulation. This protective role reduces the interfacial resistance while still allowing Li-ion transport across the buried interface.

Figure 1e presents the calculated lithium vacancy formation energies (*E*_V_) for Al-doped LATP (*x* = 0.33) and LiF.^47^ In the LATP structure, substitution of Ti with Al perturbs charge neutrality, necessitating the incorporation of two compensating lithium atoms, which occupy the 18e sites.^48^

The calculated *E*_V_ values are 4.68 and 4.69 eV for the 18e sites, whereas the 6b sites exhibit a broader distribution (5.39, 4.68, 4.70, 5.39, 5.17, and 5.18 eV). The lower formation energies at the 18e sites indicate a higher lithium chemical potential and reduced site stability, which collectively facilitate lithium migration and enhance the ionic conductivity of LATP. These results confirm that increasing the Al content introduces additional lithium sites with high chemical potential, as reflected by low *E*_V_ values, thereby creating energetically favorable pathways for Li-ion transport.

In contrast, LiF exhibits significantly higher lithium-vacancy formation energies at its 4a sites (∼6.99 eV), indicating that Li is strongly stabilized in the LiF lattice and that spontaneous vacancy formation in bulk-like LiF is energetically unfavorable. This supports the chemical stability and electronically insulating character of the LiF layer. In the ultrathin interfacial models considered here, Li transport should therefore be interpreted as interfacial or defect-assisted mobility rather than as evidence of facile bulk-like vacancy transport through LiF.

These bulk-level insights set the reference for understanding how the LiF orientation modulates band alignment, charge transfer, and transport at explicit LiF/LATP interfaces.

### 3.2 LiF/LATP Interface PDOS: (100) vs (110)

The layer-resolved PDOS results for the LiF(100)/LATP(012) heterostructure are presented in Figure 2. The LiF-side layers (first and second) retain wide band gaps of ∼6.84 and ∼6.87 eV, consistent with bulk LiF,^45,49^ indicating that the interface has little effect on deeper LiF regions. Similarly, the LATP-side layers (fourth and fifth) exhibit band gaps of ∼1.60 and ∼1.65 eV, closely matching bulk LATP,^18^ confirming that the intrinsic electronic structure of LATP is preserved in the heterostructures.

**Figure 2:**
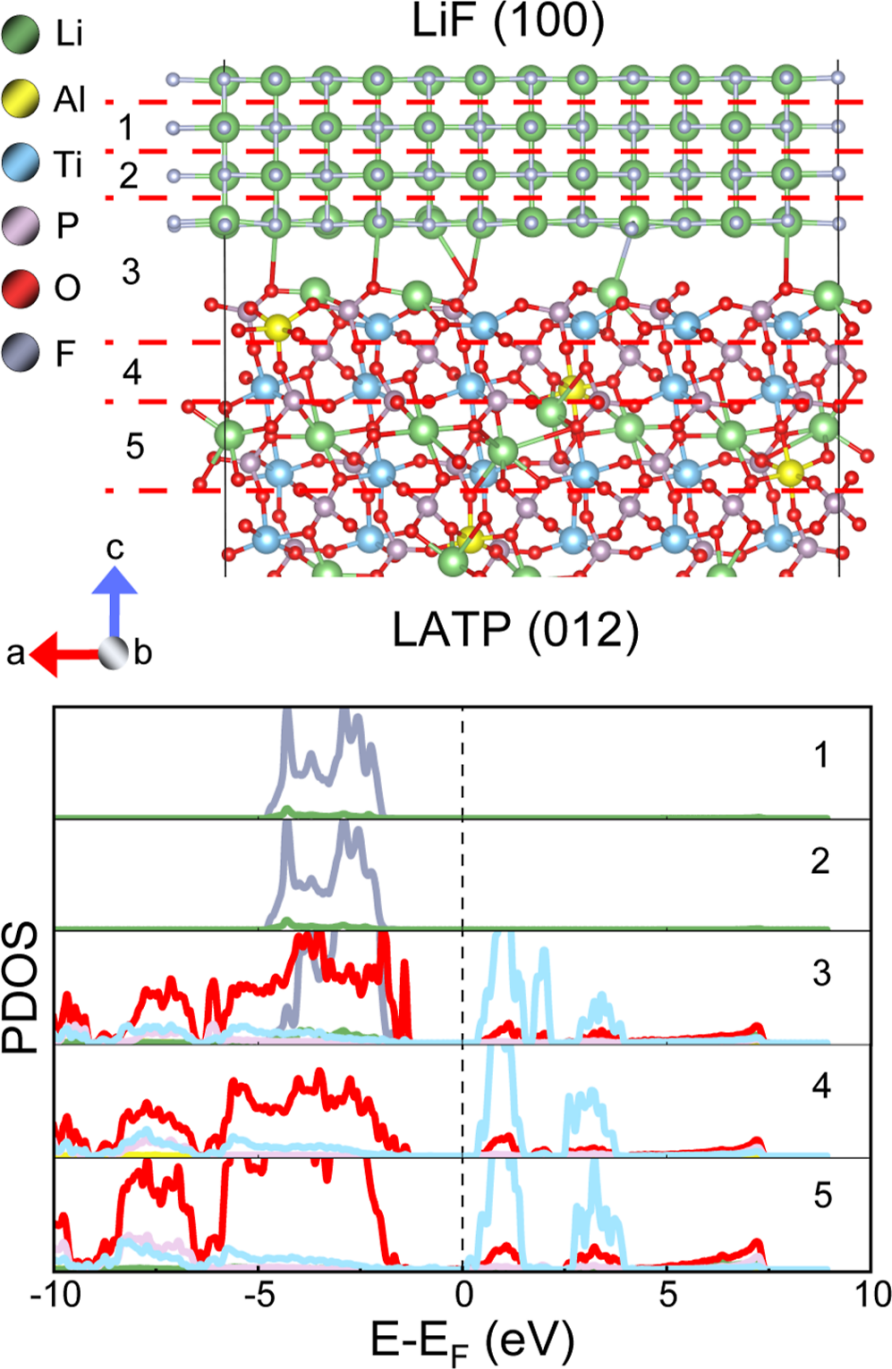
Layer-resolved PDOS of the relaxed LiF(100)/LATP(012) heterostructure. The energy axis is referenced to the Fermi level of the interface supercell. Layers 1–2 correspond to the LiF region, layer 3 represents the interfacial layer, and layers 4–5 correspond to bulk-like LATP layers. Different colors indicate atomic contributions from the corresponding layers across the interface. The absence of localized electronic states indicates suppressed electronic leakage at the interface.

In contrast, the interfacial (third) layer displays clear evidence of hybridization, with electronic states contributed by both LATP and LiF atoms.^50^ Importantly, no significant interface-induced localized electronic states appear, which would otherwise manifest as sharp localized peaks in the PDOS.^51^ Their absence indicates that the LiF(100)/LATP(012) interface is electronically stable and unlikely to promote parasitic localized charge accumulation or electron leakage.

To gain deeper insights into the interfacial electronic properties of the LiF(110)/LATP(012) heterostructure, a layer-resolved PDOS analysis was performed after geometric relaxation (Figure 3). The first and second layers, corresponding to the LiF region, retain wide band gaps of approximately 7.18 and 6.12 eV, consistent with the insulating nature of bulk LiF.^45,49^ In contrast, the fourth and fifth layers, representing bulk-like LATP, display band gaps of about 1.92 and 1.83 eV, in close agreement with the reported LATP band gap (∼2.3 eV).^18^

**Figure 3:**
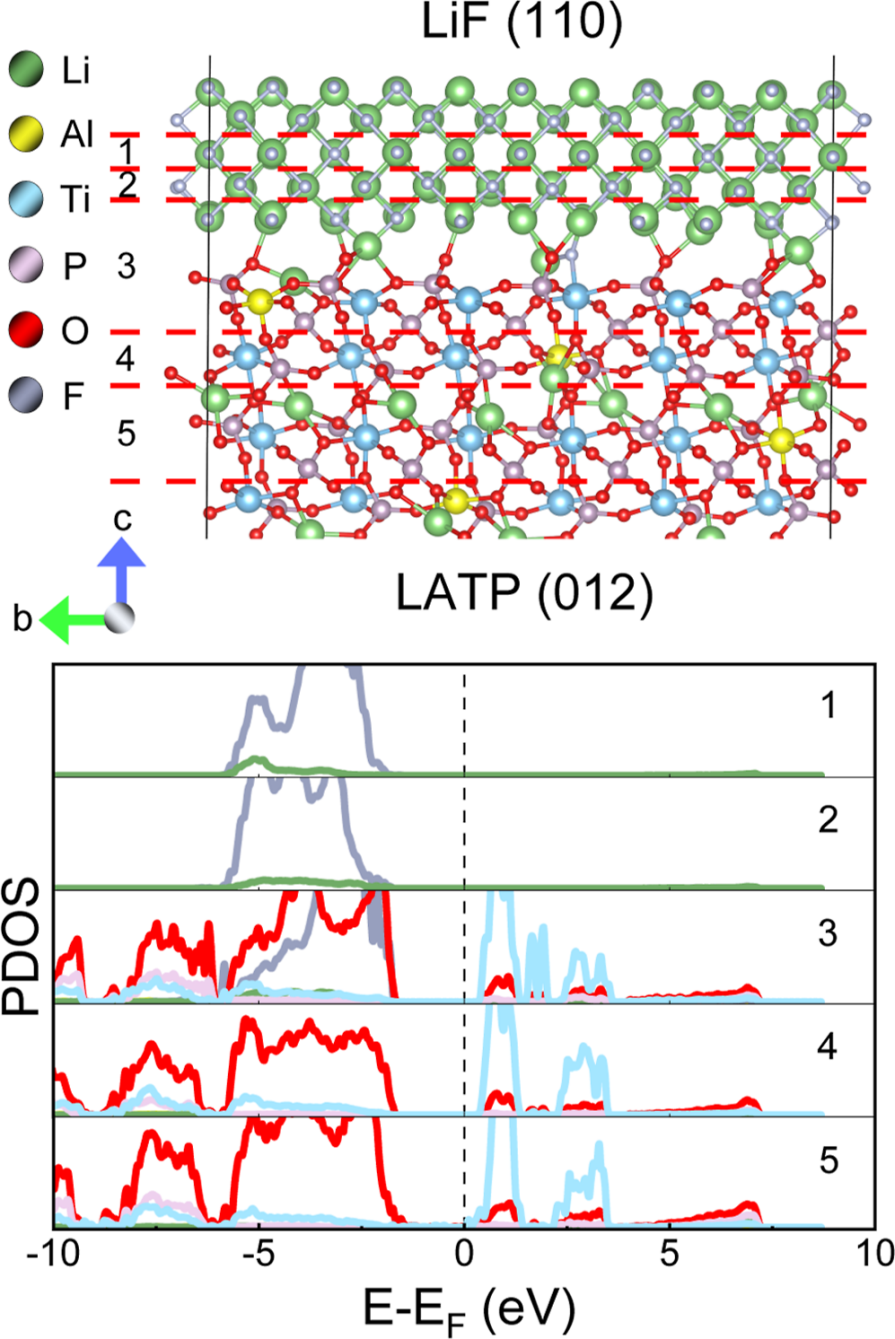
Layer-resolved PDOS of the relaxed LiF(110)/LATP(012) heterostructure. The energy scale is referenced to the Fermi level of the full interface supercell. Layers 1–2 correspond to the LiF region, layer 3 denotes the interfacial region, and layers 4–5 represent bulk-like LATP layers away from the interface. The colored PDOS curves show the atomic contributions from each layer across the heterostructure. The presence of localized interfacial states indicates stronger interfacial hybridization in the LiF(110)/LATP(012) heterostructure and a greater tendency toward electronic leakage pathways compared with the LiF(100)/LATP(012) interface.

Upon structural optimization, significant rearrangements were observed in the interfacial layers, including the formation of Li–O and Li–F bonds with optimized bond lengths of 2.60 and 2.34 Å, respectively, which compensate local electronic imbalance. Similar to the LiF(100)/LATP(012) system, the interfacial (third) layer exhibits hybrid states arising from orbital overlap between LiF and LATP atoms, confirming mixed bonding at the interface.^50^ However, unlike the (100)-oriented system, the PDOS of the interfacial layer reveals localized electronic states within 1.78–2.03 eV, a characteristic of interface-induced localized electronic states caused by strong interlayer interactions.^51^ These states indicate the possibility of deeper electron penetration and current leakage across the interface.

The comparative study of LiF(100)/LATP(012) and LiF(110)/LATP(012) heterostructures demonstrates that the crystallographic orientation of LiF strongly influences the interfacial electronic behavior. The LiF(100)/LATP(012) interface exhibits more favorable electronic insulation with no pronounced localized interfacial electronic states in the PDOS, thereby limiting parasitic electronic leakage. In contrast, the LiF(110)/LATP(012) interface shows localized interfacial electronic states that may facilitate electronic leakage, suggesting that thicker or structurally modified LiF layers may need to be considered when the (110) orientation dominates.

A zoomed-in view of the PDOS is provided in the Supporting Information (Figure S2) to further clarify the presence or absence of interface-induced states relevant to electronic leakage pathways.

### 3.3 Interfacial Charge Redistribution: Bader, Charge-Density-Difference, and ELF Analyses

To obtain a broader understanding of interfacial charge-transfer behavior and its role in suppressing Ti reduction in LATP, Bader charge analysis, CDD mapping, and ELF analysis were examined together. These descriptors provide complementary quantitative and spatial information about charge redistribution across the LiF/LATP interfaces and enable a consistent comparison between different crystallographic orientations under both neutral and negatively charged conditions.^17,52^

Bader charge analysis was first performed to evaluate the sensitivity of Ti-centered sites to excess electronic charge in the LiF(100)/LATP(012), LiF(110)/LATP(012), and LiF(111)/LATP(012) heterostructures.^53^ As shown in Figure 4, across all orientations, the calculated Bader charge transfer (Δ*q*_*i*_ = *q*_*i*_^ref^ – *q*_*i*_^Bader^) of Ti atoms remains within a narrow range (≈1.93–2.06 e) in both neutral and negatively charged configurations, indicating that the introduction of excess electrons does not lead to significant charge accumulation on Ti-centered octahedra. Importantly, the variations observed between neutral and charged systems are small and comparable to typical redistribution effects associated with interfacial polarization rather than changes in the formal oxidation state. Because Bader charge transfer does not directly correspond to formal oxidation states and variations on the order of ∼0.05–0.1 e fall within the typical uncertainty range of GGA-level DFT calculations, it is interpreted here as indicators of relative charge redistribution rather than absolute measures of Ti valence. Within this framework, the nearly unchanged Ti charge transfer suggests that electron penetration into the LATP framework is limited by the LiF interlayer.^43^ The full Ti charge-transfer values for all neutral and negatively charged heterostructures are provided in the Supporting Information (Table S2).

**Figure 4:**
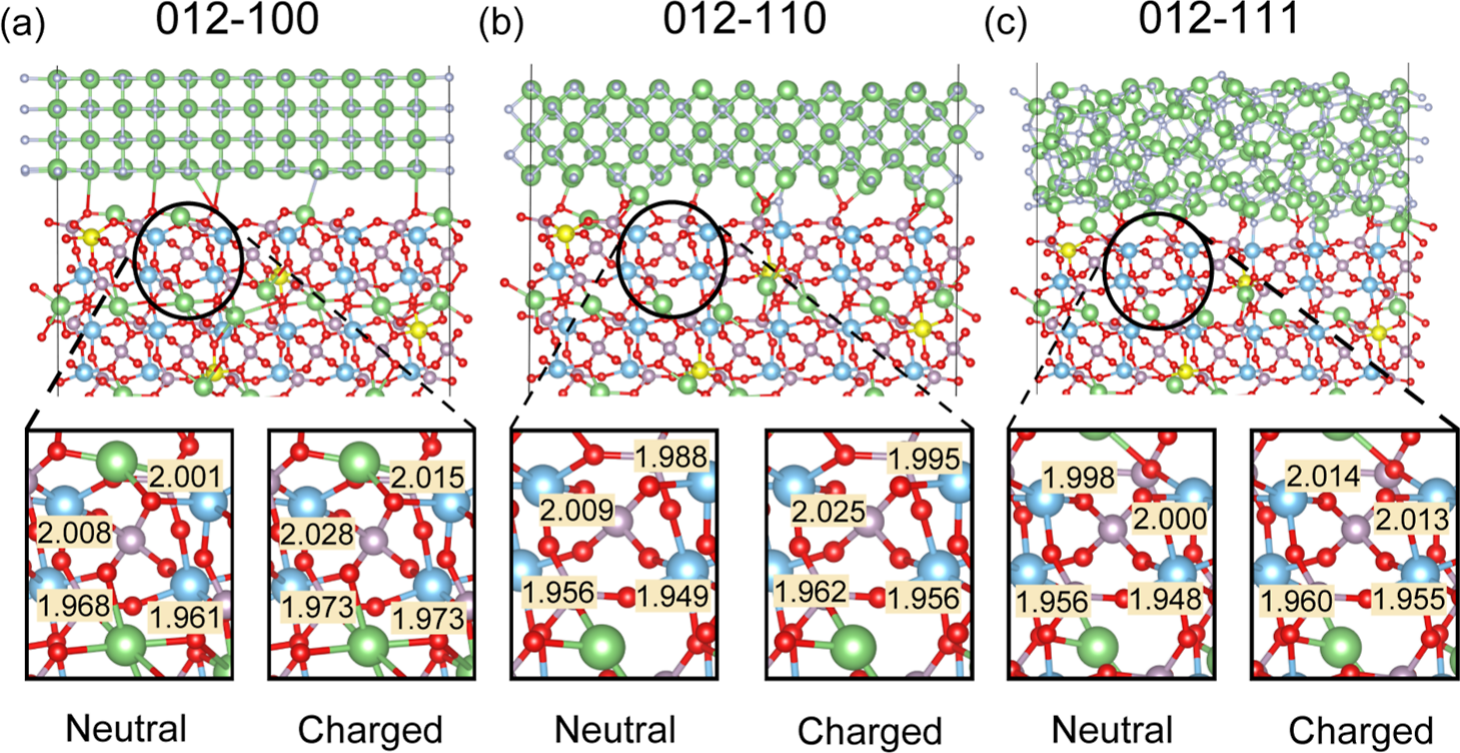
Bader charge transfer of Ti atoms in (a) LiF(100)/LATP(012), (b) LiF(110)/LATP(012), and (c) LiF(111)/LATP(012) heterostructures under neutral and negatively charged conditions. Across all orientations, Ti exhibits minimal charge variation, indicating that the LiF interlayer limits electron transfer into Ti-centered sites and suppresses significant Ti-centered charge accumulation. The LiF(100)/LATP(012) interface shows the smallest change upon charge injection, consistent with its more effective electronic blocking behavior.

To further resolve the spatial distribution of interfacial charge transfer, CDD analysis was performed for all heterostructures.^51^ The CDD is defined as

2 $$\Delta \rho \left(r\right)=\rho_{LiF/LATP}\left(r\right)-\rho_{LATP}\left(r\right)-\rho_{LiF}\left(r\right)$$

where the reference densities correspond to isolated slabs computed within the same supercell geometry. The resulting CDD maps shown in Figure 5 reveal localized regions of electron accumulation and depletion concentrated near the LiF/LATP interface.^54^ To enable a direct comparison of interfacial charge redistribution across different heterostructures, Bader-derived charge transfers (Δ*Q*) were normalized by the respective interface area (*A*), yielding the area-normalized charge redistribution, Δ*Q*_norm_ = Δ*Q*/*A*. The normalized values are reported in *e* nm^–2^ and are shown together with the CDD isosurfaces in Figure 5.

**Figure 5:**
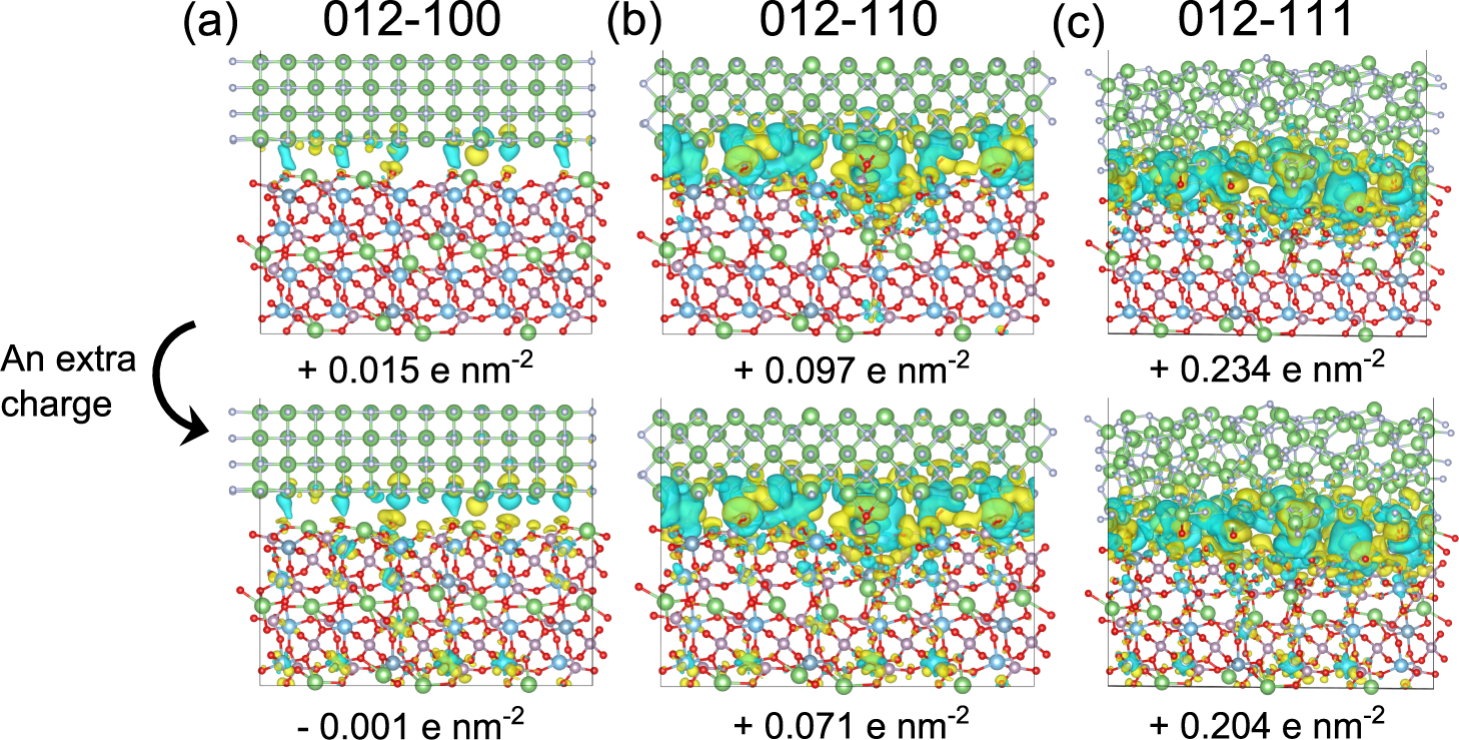
Charge-density-difference (CDD) isosurfaces (0.0007 e Å^–3^) together with Bader charge-transfer analysis across LiF/LATP interfaces with different crystallographic orientations: (a) LiF(100)/LATP(012), (b) LiF(110)/LATP(012), and (c) LiF(111)/LATP(012). For each orientation, the upper and lower panels correspond to neutral and excess-charge conditions, respectively, where the excess-charge state contains one additional electron per supercell. Yellow and cyan regions represent electron accumulation and depletion, respectively. The reported charge-redistribution values are normalized by the LiF/LATP interface area, Δ*Q*_norm_ = Δ*Q*/*A*, and are given in *e* nm^–2^. The LiF(100)/LATP(012) interface exhibits a comparatively lower area-normalized charge redistribution, whereas the LiF(111)/LATP(012) interface shows a larger normalized charge redistribution, consistent with stronger interfacial electronic coupling inferred from the PDOS and ELF analyses.

Among the three orientations, the LiF(100)/LATP(012) configuration exhibits the smallest area-normalized interfacial charge redistribution, indicating weaker electronic coupling and a smaller and more spatially confined charge redistribution. In contrast, the LiF(110)/LATP(012) and LiF(111)/LATP(012) interfaces show larger normalized charge redistribution across the interface region, consistent with a stronger interfacial electronic interaction.^55,56^

To evaluate the response of the interfaces to an additional electronic charge, the CDD analysis was repeated after introducing one excess electron into each heterostructure, as illustrated in Figure 5. In all cases, the additional charge remains primarily localized near the interfacial region rather than accumulating on Ti-centered sites in the LATP framework. This spatial confinement suggests that the LiF interlayer helps limit electron transfer into the LATP lattice even under negatively charged conditions. The orientation-dependent differences observed in the spatial extent of charge redistribution further support the interpretation that the LiF(100) interface may provide the most favorable electronic blocking behavior among the configurations considered.

Although the absolute magnitude of the charge density differences between neutral and charged configurations is relatively small, their spatial distribution and systematic dependence on the LiF crystallographic orientation provide meaningful insights into interfacial electronic coupling. In particular, the reduced charge transfer observed for the LiF(100)/LATP(012) interface is consistent with its weaker interfacial polarization and smaller dipole moment, whereas the larger redistribution observed for the LiF(110) and LiF(111) orientations reflects a stronger electronic interaction across the interface. Therefore, the significance of the CDD results lies primarily in their orientation-dependent trends rather than in their absolute numerical magnitudes.

Additional insights into the bonding character at the interface were obtained from ELF analysis. The corresponding ELF maps shown in Figure 6 further clarify the orientation-dependent nature of the interfacial electronic interaction. It should be noted that the ELF provides a qualitative measure of electron localization and does not constitute direct evidence of covalent bonding. Accordingly, the variations observed here are interpreted in terms of changes in interfacial electronic overlap and orbital hybridization rather than definitive changes in the bonding character.

**Figure 6:**
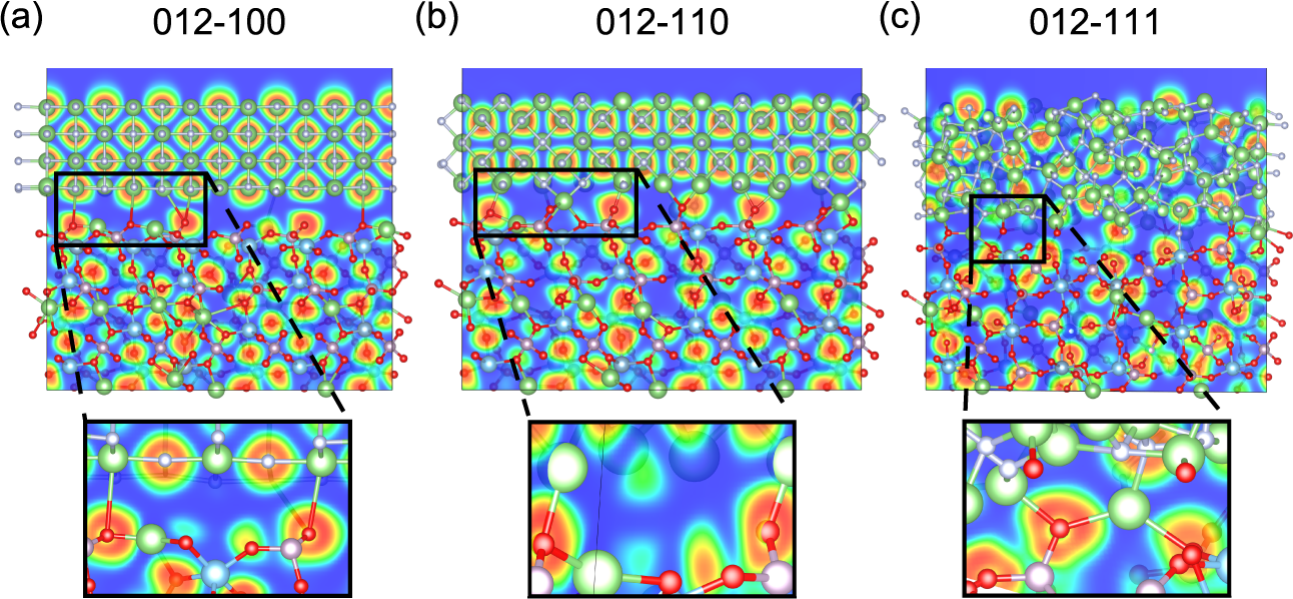
Two-dimensional electron localization function (ELF) maps for LiF/LATP interfaces: (a) LiF(100)/LATP(012), (b) LiF(110)/LATP(012), and (c) LiF(111)/LATP(012). Upper panels show cross-sectional slices through the center of each heterostructure, while lower panels highlight magnified interfacial bonding regions. Red and blue indicate high- and low-electron localization, respectively. The interfacial region shows progressively stronger electron localization from (100) to (111), suggesting increased electronic interactions and charge redistribution, in line with the CDD and PDOS analyses.

In the LiF region, the ELF distribution confirms the predominantly ionic nature of Li–F bonding, with strong electron localization around F atoms and depletion near Li atoms. In the LATP framework, elevated ELF values near oxygen atoms indicate polar covalent P–O bonding, while the Li sites retain their ionic character. At the LiF/LATP interface, the ELF maps reveal a clear orientation dependence in the degree of electronic overlap between adjacent layers. The LiF(100)/LATP(012) configuration exhibits minimal interfacial electron localization, indicating weak orbital overlap and predominantly ionic interaction across the interface. By contrast, the LiF(110)/LATP(012) and LiF(111)/LATP(012) configurations show increased interfacial electron localization, consistent with stronger electronic interactions at the interface and enhanced charge redistribution identified in the CDD analysis.

Taken together, the combined Bader, CDD, and ELF analyses suggest an orientation-dependent pattern of interfacial charge redistribution in LiF/LATP heterostructures. The absence of systematic charge accumulation on Ti-centered sites, together with the spatial confinement of excess electrons near the interface, indicates that the LiF interlayer can help limit electron penetration into LATP. Among the configurations investigated, the LiF(100)/LATP(012) interface exhibits the weakest interfacial charge transfer and the most localized redistribution, which is consistent with more favorable electron blocking behavior relative to the higher index orientations.

### 3.4 Electrostatic Origin of Orientation-Dependent Interfacial Stability

#### 3.4.1 Interface Dipole Moments and Electrostatic Polarization

To characterize the electrostatic perturbations arising from the LiF/LATP interface formation, the interfacial dipole moment, μ_*z*_, was calculated by evaluating the first moment of the planar-averaged charge density difference (CDD) profile.^57^ The three-dimensional charge-density difference, Δρ­(*x*, *y*, *z*), was first averaged over the interfacial plane, i.e., the *xy*-plane, to yield the planar-averaged profile, Δρ­(*z*)­

3 $$\Delta \rho \left(z\right)=\frac{1}{A}\iint_{A}\Delta \rho \left(x,y,z\right)\;dxdy$$

where *A* represents the area of the interfacial cell. The interfacial dipole moment, μ_*z*_, was subsequently derived by integrating this profile along the surface-normal direction

4 $$\mu_{z}=\int \left(z-z_{0}\right)\Delta \rho \left(z\right)\;dz$$

where *z*_0_ denotes the reference plane located at the geometrical LiF/LATP interface, maintained consistently across all computational models. Given that Δρ­(*z*) is expressed in units of *e*/Å^3^, the resulting μ_*z*_ carries the units of *e*/Å. This metric serves as a quantitative descriptor of the interfacial polarization, thereby facilitating a comparative analysis of charge redistribution across diverse interface architectures.

The calculated interfacial dipole moments exhibit a clear orientation dependence. Among the investigated configurations, the LiF(100)/LATP(012) interface shows the smallest dipole moment, indicating a relatively weak interfacial polarization and limited charge redistribution. In contrast, the LiF(110)/LATP(012) configuration develops the largest dipole moment, consistent with a stronger interfacial charge rearrangement. The LiF(111)/LATP(012) interface shows a sizable dipole moment, reflecting the influence of its partially polar surface termination and interfacial coupling.^58^

Importantly, the interfacial dipole moment and the built-in electric field originate from the same underlying interfacial charge-redistribution processes, although they describe different aspects of the electrostatic response. While the dipole moment represents an integrated measure of charge asymmetry across the interface, the local electric field is given by the spatial derivative of the electrostatic potential

5 $$E_{z}=-\frac{dV\left(z\right)}{dz}$$

In general, larger dipole moments may be associated with stronger electrostatic perturbations, although the local field profile also depends on the spatial distribution and screening of charge.^58^ In this respect, the LiF(110)/LATP(012) interface exhibits both the largest dipole moment and the most pronounced field inhomogeneity, whereas the LiF(100)/LATP(012) configuration shows a much smaller dipole moment together with a weaker and more uniform electric field distribution. By contrast, the LiF(111)/LATP(012) interface shows a sizable dipole moment, but its field distribution is more localized and spatially complex, indicating that the net dipole moment magnitude alone does not fully determine the detailed field profile.

Overall, these results support the interpretation that the orientation-dependent dipole response is closely linked to the extent of interfacial charge redistribution revealed by the planar-averaged charge density difference profiles. In particular, the reduced dipole moment observed for the LiF(100)/LATP(012) configuration is consistent with a weaker electrostatic perturbation and potentially reduced electronic leakage compared to the higher index LiF orientations.

In all three interface models, the LiF interlayers consist of approximately 3–5 atomic layers with a nearly identical thickness of about 6 Å (5.88–5.99 Å; <3% variation). Although this fixed thickness does not aim to represent the full experimental range of LiF interlayers, it is physically reasonable for an ultrathin protective layer, where interfacial electronic effects remain relevant while the LiF slab largely retains its insulating character away from the interface.

Experimental LiF-based interlayers on LATP can be substantially thicker; for example, sputtered LiF interlayers with a thickness of approximately 50 nm have been reported for LiF-modified LATP. Such experimental length scales are beyond the tractable thickness range of the present first-principles slab model. The purpose of the present calculations is therefore not to determine the optimum LiF thickness but to isolate how crystallographic orientation affects a local interfacial electronic structure, charge redistribution, electrostatic polarization, and Li coordination in ultrathin LiF/LATP interface models.^24^

Accordingly, the conclusions drawn here should be interpreted for ultrathin LiF interlayers of comparable thickness rather than for all possible LiF thicknesses.

#### 3.4.2 Interfacial Electric-Field Response

Figure 8 provides a real-space view of the electrostatic response through two-dimensional maps of the out-of-plane electric field across the LATP/LiF interfaces.^59,60^ The LiF(100)/LATP(012) heterostructure exhibits a uniform and smoothly modulated electric field distribution with moderate intensity, indicative of efficient electrostatic screening and weak interfacial polarization. This homogeneous field profile is fully consistent with the minimal charge transfer and the smaller interface dipoles identified in Figure 7.

**Figure 7:**
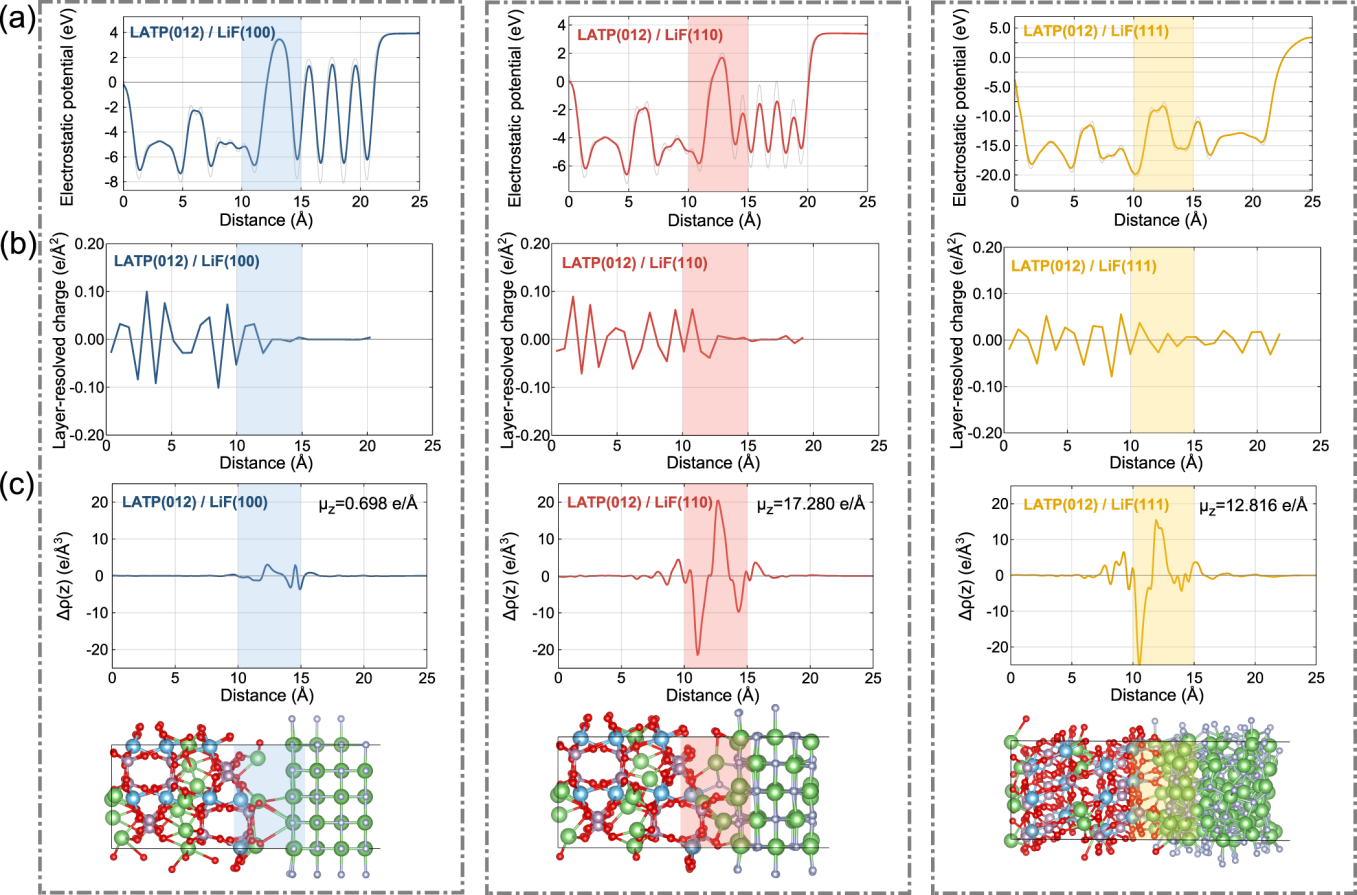
Electrostatic characteristics of LATP(012)/LiF interfaces with different LiF crystallographic orientations: (left) LiF(100), (middle) LiF(110), and (right) LiF(111). (a) Macroscopic electrostatic potential profiles across the interface, (b) layer-resolved charge variation along the interface normal, and (c) planar-averaged charge-density-difference profiles, $\overset{®}{\Delta \rho }\left(z\right)$ , together with the corresponding interface dipole moments, μ_*z*_, obtained from their first moments. The LiF(100) orientation shows more localized interfacial charge redistribution and a smaller μ_*z*_, indicating weaker interfacial polarization, more effective electrostatic screening, and more favorable electronic insulation relative to the LiF(110) and LiF(111) orientations considered here.

**Figure 8:**
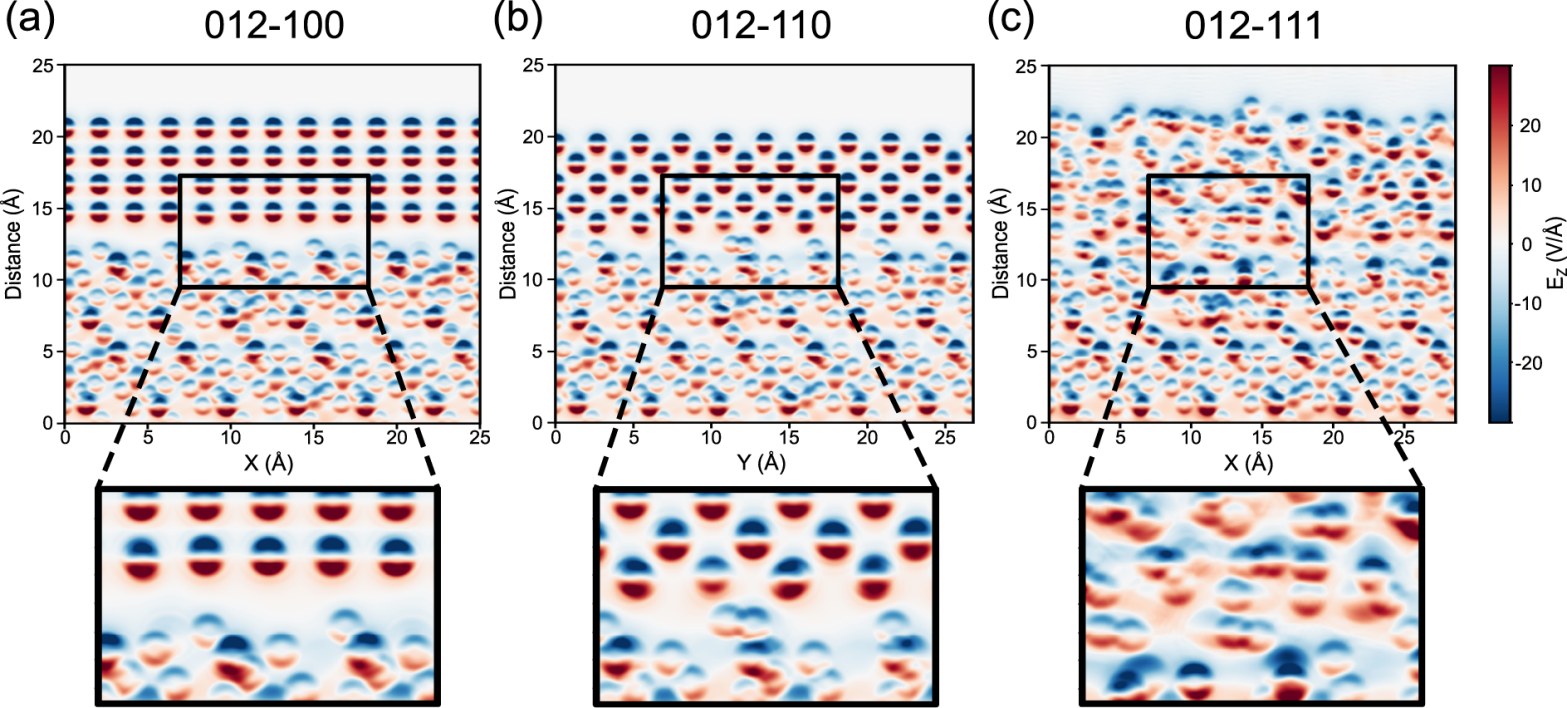
Two-dimensional maps of the out-of-plane electric field *E*_*z*_ across LiF/LATP interfaces with different crystallographic orientations: (a) LiF(100)/LATP(012), (b) LiF(110)/LATP(012), and (c) LiF(111)/LATP(012). Upper panels show the electric-field distributions across the interfaces, while lower panels present magnified views of the interfacial regions. The LiF(100)/LATP(012) interface exhibits a more uniform and well-screened electric-field distribution, whereas the (110) and (111) orientations show increasingly localized and inhomogeneous fields, indicating stronger interfacial polarization and enhanced electronic coupling.

In contrast, the LiF(110)/LATP(012) interface displays pronounced spatial fluctuations in the electric field, including localized regions of an enhanced intensity near the interface. These inhomogeneities reflect stronger interfacial polarization and reduced screening efficiency, which correlate with the emergence of interfacial electronic states. The LiF(111)/LATP(012) system shows the highest degree of field localization and spatial disorder, signaling a strong electronic hybridization and substantial charge redistribution.

The electric-field maps provide electrostatic evidence that the crystallographic orientation strongly influences charge screening at the LiF/LATP interface. The more uniform and better screened electric field associated with the LiF(100) orientation supports its more favorable electron-blocking behavior, whereas higher index orientations induce more localized fields that may enhance electronic coupling and interfacial polarization.

### 3.5 Adhesion Energies

Adhesion energies were calculated to quantify the interfacial binding strength of the LiF/LATP heterostructures according to

6 $$E_{adh}=\frac{E_{LiF/LATP}-\left(E_{LiFslab}+E_{LATPslab}\right)}{A}$$

where *E*_interface_ is the total energy of the relaxed LiF/LATP heterostructure, and *E*_LiF slab_ and *E*_LATP slab_ are the total energies of the corresponding isolated slabs obtained from the same supercell after removing the counterpart slab, without further structural modification, and computed using identical numerical parameters, including vacuum thickness (20 Å), *k*-point sampling, and plane-wave cutoff.

The interface models were constructed as asymmetric slab geometries containing a single LiF/LATP interface; therefore, normalization by *A* was applied. Dipole corrections were included along the surface-normal direction to remove artificial electrostatic interactions associated with the asymmetric slab geometry. Within this sign convention, more negative *E*_adh_ values correspond to stronger interfacial binding and more favorable thermodynamic adhesion. Typical adhesion energies reported for oxide and ceramic solid–solid interfaces range from approximately 0.1 to a few Jm^–2^, while strongly bonded ionic heterointerfaces may exhibit larger magnitudes.

Although the LiF(100)/LATP(012) interface exhibits a lower adhesion than the LiF(110)/LATP(012) and LiF(111)/LATP(012) interfaces, this behavior is not detrimental to its role as an electronic insulating protective interlayer. At solid-state battery interfaces, excessively strong adhesion can, in some cases, be accompanied by increased electronic hybridization across the interface, which may promote interfacial reduction reactions and the formation of electronic leakage pathways. In contrast, the LiF(100)/LATP(012) orientation shows reduced interfacial charge transfer, a smaller interfacial dipole moment, and the absence of interface-induced localized electronic states, indicating suppressed electronic coupling between LiF and LATP. From this perspective, the moderate adhesion of this configuration represents a favorable balance between the mechanical stability and electronic insulation.

### 3.6 Ab Initio Molecular Dynamics Analysis

To further evaluate the short-time scale thermal and structural stability of the LiF/LATP heterostructures with different LiF orientations, AIMD simulations were performed in the canonical (*NVT*) ensemble at 300 K for a total simulation time of 20 ps, as illustrated in Figure 9. The first 5 ps were treated as equilibration and were excluded from subsequent analysis.

**Figure 9:**
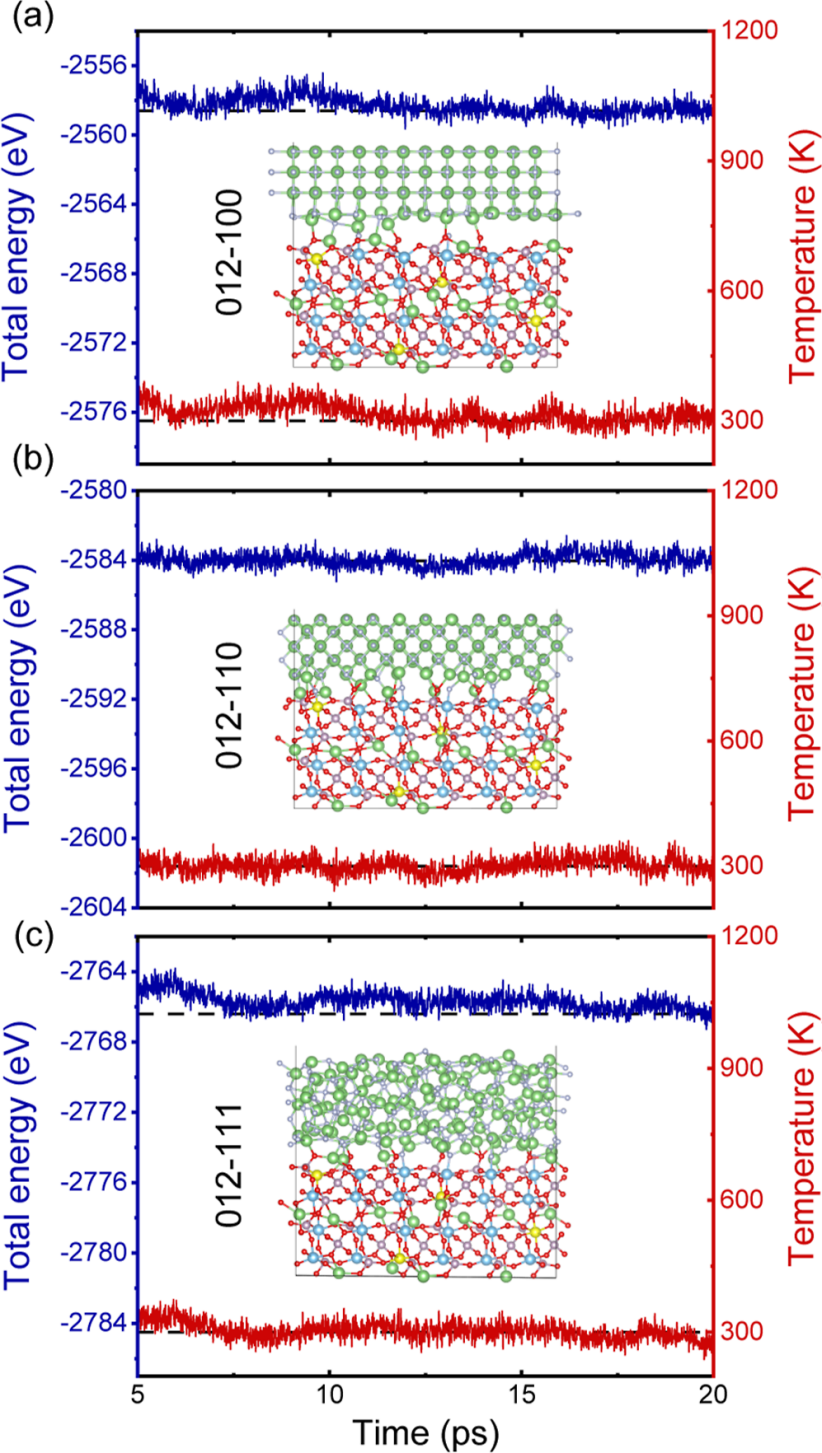
Fluctuations of the total energy (blue) and temperature (red) during AIMD simulations of (a) LiF(100)/LATP(012), (b) LiF(110)/LATP(012), and (c) LiF(111)/LATP(012) heterostructures performed in the *NVT* ensemble at 300 K. Representative snapshots taken from the final AIMD configurations are shown in each panel. The first 5 ps were treated as equilibration and excluded from analysis. The small stationary fluctuations indicate stable thermal regulation and dynamical behavior for all interface orientations within the simulated time scale.

During the production stage, the temperature fluctuated smoothly around the target value, with standard deviations of 22.19, 17.69, and 19.24 K for the 012–100, 012–110, and 012–111 heterostructures, respectively, confirming stable thermal regulation throughout the trajectories.

Likewise, the total energies remained nearly constant after equilibration, showing only small fluctuations with standard deviations of 0.46, 0.37, and 0.49 eV, indicating a stable dynamical behavior within the simulated time window.

No evidence of interfacial delamination or structural degradation was observed during the 20 ps AIMD simulations at 300 K, indicating that all heterostructures remain structurally stable within the simulated time scale. The slightly smaller fluctuations observed for the 012–110 configuration suggest marginally enhanced dynamical stability compared with the other orientations, although all interfaces exhibit stable structural behavior over the duration of the simulations.

### 3.7 Correlating Li Diffusion with Interfacial Atomic Ordering

To quantify how interfacial atomic arrangements regulate lithium transport, Li^+^ mobility across different LiF/LATP orientations was evaluated using mean-square displacement (MSD) analyses. The MSD, averaged over all Li ions and multiple time origins, is defined as

7 $$\left\langle r^{2}\left(t\right)\right\rangle =\frac{1}{N}\sum_{i=1}^{N}\left\langle {\left|\vec{r_{i}}\left(t\right)-\vec{r_{i}}\left(0\right)\right|}^{2}\right\rangle$$

and diffusion coefficient *D* is extracted from the long-time linear regime of the MSD using the Einstein relation

8 $$D=\lim_{t\to \infty }\frac{MSD\left(t\right)}{2dt}$$

where *d* is the dimensionality of the diffusion analysis. For the apparent three-dimensional MSD used here, *d* = 3.

To evaluate the convergence of the extracted diffusion coefficients, linear fits to the MSD curves were performed over multiple independent time windows within the diffusive regime (5–20 ps, 10–20 ps, and 15–20 ps). The resulting diffusion coefficients differ by only ∼2%, confirming that the slope of the long-time MSD region is stable with respect to the selected fitting interval. This variation provides an estimate of the statistical uncertainty associated with the finite simulation time and demonstrates that the reported diffusion coefficients are robust within the accessible AIMD time scale. The MSD profiles shown in Figure 10a reveal a clear orientation-dependent lithium mobility at the LiF/LATP interface. Among the evaluated configurations, the LiF(100)/LATP(012) heterostructure exhibits the lowest apparent diffusion coefficient (1.48 × 10^–4^ cm^2^ s^–1^), consistent with its weaker interfacial electronic coupling and limited charge redistribution. It should be noted that the reported diffusion coefficients primarily reflect lithium motion within the LiF interfacial layer and the adjacent LATP surface region rather than bulk LATP diffusion. This distinction is explicitly considered when interpreting the transport mechanism across the heterostructure interfaces. In contrast, the LiF(110)/LATP(012) interface shows the highest mobility (2.78 × 10^–4^ cm^2^ s^–1^), reflecting stronger interfacial interactions that correlate with more favorable migration pathways. The LiF(111)/LATP(012) orientation provides an intermediate diffusivity (2.15 × 10^–4^ cm^2^ s^–1^), in agreement with its moderate degree of interfacial hybridization.^61^ It should be emphasized that diffusion coefficients extracted from relatively short AIMD trajectories (20 ps at 300 K) are not expected to represent fully converged transport parameters. Instead, the reported values primarily provide a comparative measure of orientation-dependent lithium mobility across the interfaces. Therefore, the most physically meaningful outcome of the present analysis is the robust trend

$$D_{100}<D_{111}<D_{110}$$

rather than the absolute magnitude of the diffusion coefficients themselves.

**Figure 10:**
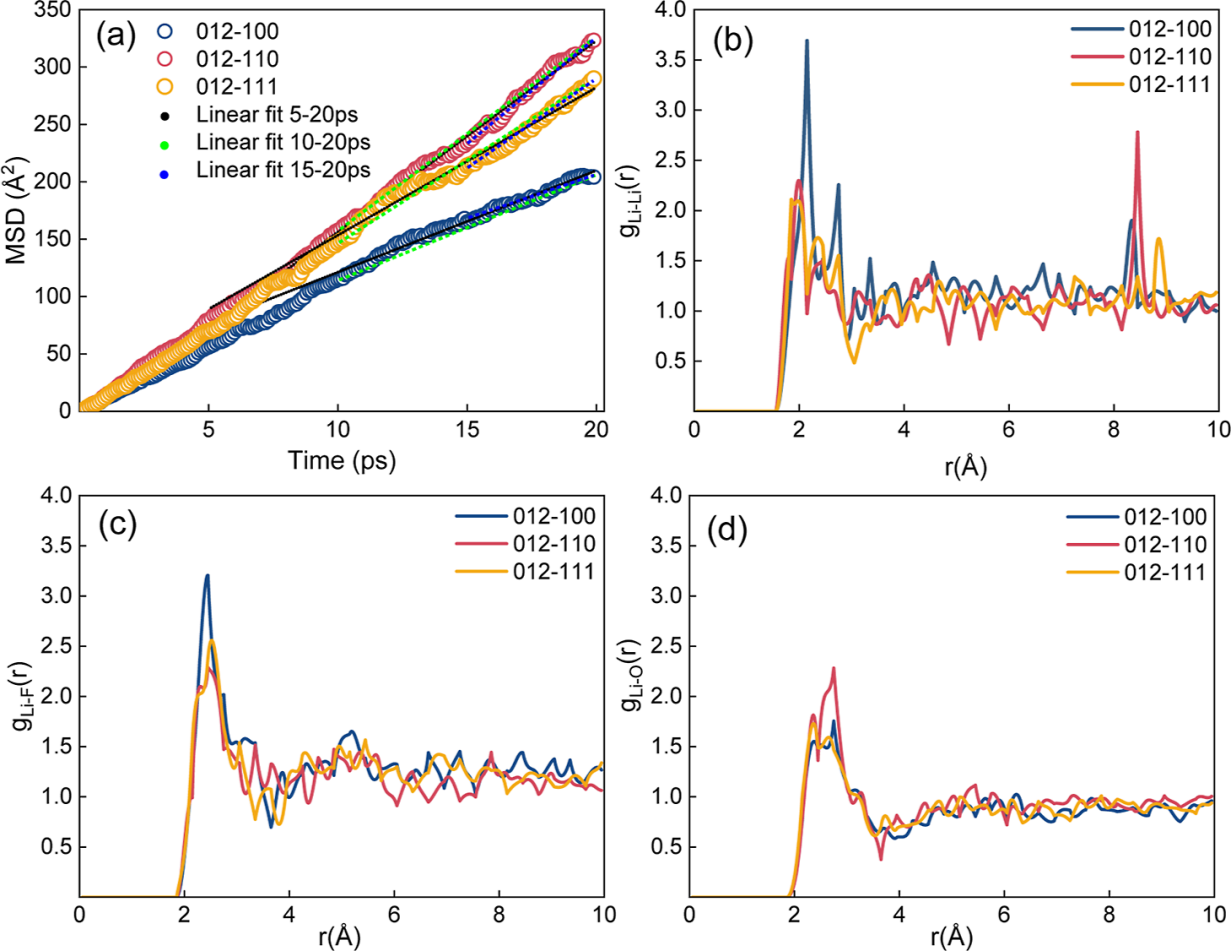
(a) Mean-square displacement (MSD) profiles of Li ions for the LiF(100)/LATP(012), LiF(110)/LATP(012), and LiF(111)/LATP(012) heterostructures at 300 K, yielding an orientation-dependent diffusivity trend *D*_100_ < *D*_111_ < *D*_110_. The MSD values are plotted in Å^2^, and the diffusion coefficients were calculated after converting the fitted slopes to cm^2^ s^–1^. (b) Li–Li radial distribution function (RDF) with a pronounced peak at 2.15 Å, strongest for 012–100, indicating more ordered Li environments. (c) Li–F RDF with a main peak at 2.35 Å, reflecting orientation-dependent Li–F coordination strength. (d) Li–O RDF with a peak near 2.55 Å, weakest for 012–100 and strongest for 012–110, consistent with enhanced Li–O interactions and higher Li mobility at higher index interfaces.

The radial distribution function (RDF) analysis further clarifies how an interfacial orientation shapes the local coordination environment governing Li diffusion. The Li–Li RDF peak at 2.15 Å reaches its highest intensity (3.69) for the 012–100 heterostructure, indicating stronger Li–Li correlations and a more ordered lithium environment—consistent with its reduced diffusivity (Figure 10b). The 012–110 and 012–111 heterostructures exhibit lower peak heights of 2.68 and 2.59, respectively. A similar orientation-dependent trend appears in the Li–F correlations at 2.35 Å, where peak intensities decrease from 3.24 (012–100) to 2.38 (012–111) and 2.28 (012–110), highlighting variations in Li–F coordination (Figure 10c).

Conversely, the Li–O RDF peak at 2.55 Å is the lowest for 012–100 (1.67) and highest for 012–110 (2.37), indicating enhanced Li–O interactions at higher index interfaces (Figure 10d). These stronger Li–O correlations at the 012–110 interface are consistent with the presence of interface-induced localized electronic states and enhanced electronic hybridization inferred from the PDOS and CDD analyses.

Overall, the combined MSD and RDF analyses demonstrate that the 012–100 interface sustains more ordered and tightly coordinated local environments that restrict Li mobility, whereas higher index orientations introduce a structural disorder and modified bonding (particularly enhanced Li–O coordination) that facilitate lithium transport. Together with the PDOS and CDD results, these findings highlight that LiF(100) provides the most favorable balance between electronic insulation and controlled Li-ion transport at LATP interfaces.

To further examine the relationship between the interfacial electrostatic environment and Li mobility, Bader charge analysis for Li atoms located near the LiF/LATP interface is revisited (Figure 5). The results reveal a clear orientation dependence in the degree of charge localization around the interfacial Li ions. In particular, Li atoms at the LiF(100)/LATP(012) interface exhibit slightly stronger charge localization, consistent with their more ordered coordination environment and the reduced mobility observed in the MSD analysis. By contrast, Li atoms near the LiF(110)/LATP(012) interface show comparatively weaker charge localization, indicating an enhanced interfacial electronic interaction that facilitates Li-ion migration. The LiF(111)/LATP(012) configuration again displays an intermediate behavior. These results provide additional support for the correlation between interfacial charge redistribution and orientation-dependent Li diffusion trends. The diffusion coefficients reported here should be interpreted primarily as orientation-dependent comparative indicators of interfacial lithium mobility rather than as fully converged bulk transport parameters.

## 4 Conclusions

This work presents a first-principles and ab initio molecular dynamics investigation of how the LiF crystallographic orientation influences the structural, electronic, and ionic properties of the ultrathin LiF/LATP(012) heterostructures considered here. Bulk analyses confirm LATP as a wide-band gap, fast-ion-conducting NASICON electrolyte and LiF as an electronically insulating phase with a high lithium-vacancy formation energy. The resulting chemical potential contrast supports Li-ion transport primarily through interfacial or defect-assisted pathways, while LiF preserves electronic insulation, establishing LiF as an intrinsically suitable protective interlayer for LATP-based solid electrolytes. Across all investigated configurations, the presence of LiF appears to suppress Ti^4+^ reduction, as Bader charge analysis reveals only negligible changes in the Ti charge state, in contrast to those of bare Li/LATP interfaces.

By combining first-principles calculations with a descriptor-based framework, we systematically extracted physically motivated electrostatic and structural descriptors. Within this framework, the LiF(100)/LATP(012) interface shows a favorable interfacial response among the orientations investigated in this study, exhibiting reduced area-normalized charge redistribution, the absence of pronounced localized interfacial electronic states in the layer-resolved PDOS, weaker interfacial polarization, more ordered Li coordination, and comparatively lower Li-ion diffusion. These features collectively suggest a favorable balance between electronic blocking and controlled ionic transport within the interface models considered here.

In contrast, the LiF(110)/LATP(012) and LiF(111)/LATP(012) interfaces display enhanced electronic hybridization, stronger Li–O interactions, more pronounced interfacial polarization, and higher Li-ion mobility. The LiF(110) orientation, in particular, combines strong adhesion with the emergence of interface-induced localized electronic states within the band gap and accelerated Li diffusion, reflecting increased electronic and ionic coupling across the interface, while LiF(111) shows intermediate behavior. AIMD simulations indicate short-time scale thermal stability of all interfaces at 300 K within the duration of the simulations, with no signs of delamination or structural degradation over the simulated time scales. The contrast between lithium-site stability in LiF and the finite Li mobility observed in the ultrathin interface models supports the role of LiF as a chemically stable and electronically insulating interlayer, while lithium mobility should be interpreted primarily as interfacial or defect-assisted transport.

Overall, these results suggest that the crystallographic orientation is an important factor to consider in the design of ultrathin LiF-based protective interlayers for LATP solid electrolytes. From an application standpoint, ultrathin LiF coatings preferentially exposing the LiF(100) orientation may provide a favorable compromise between interfacial stability and regulated Li transport for LATP-based solid-state lithium–metal batteries.

Although the present analysis focuses on three representative LiF orientations on LATP(012), the descriptor-based approach adopted here can serve as a useful framework for comparing other interface terminations and protective interlayer materials in future studies.

## Supplementary Material



## Notes

Structural details of the interface models, Ti Bader charge values, representative DFT + *U* robustness check for LiF(100)/LATP(012), and PDOS analyses (PDF)
