# Supplementary material for: Orientation-Dependent Protection by LiF Interlayers at LATP Solid–Electrolyte Interfaces: A First-Principles Study
Source: ACS Appl Mater Interfaces. 2026 Jun 21;18(25):36151–63. doi: 10.1021/acsami.6c02962 (PMC13339027; doi:10.1021/acsami.6c02962)
Supplement: Supplementary file 1 [file am6c02962_si_001.pdf]

Supporting Information — Orientation-dependent protection by LiF interlayers at LATP solid-electrolyte interfaces: a first-principles study

Maryam Kookhaee,[†,‡] Ali Lashani Zand,[†,‡] Maryam Soleimani,[†] Nicola Seriani,[‡] Shamsoddin Mohajerzadeh,[†] and Mahdi Pourfath[*,†,¶]

†*School of Electrical and Computer Engineering, College of Engineering, University of Tehran, Tehran 14395-515, Iran*

‡*Condensed Matter and Statistical Physics Section, The Abdus Salam International Centre for Theoretical Physics, Strada Costiera 11, 34151 Trieste, Italy*

¶*Institute for Microelectronics, TU Wien, Gusshausstrasse 27-39/E360, 1040 Vienna, Austria*

E-mail: pourfath@tuwien.ac.at

Electrostatic Analysis of LATP/LiF Interfaces

This Supporting Information provides the detailed computational definitions and electrostatic descriptors underlying the analyses discussed in Section 3.4 of the main text. The focus is placed on the macroscopic electrostatic potential, layer-resolved charge redistribution, interface dipole moments, and the real-space interfacial electric-field response.

Planar-Averaged and Macroscopic Electrostatic Potential

The total electrostatic potential $V(x, y, z)$ was obtained from self-consistent density functional theory calculations using the `LOCPOT` output of VASP. To characterize the electrostatic response across the interface, the potential was analyzed through planar and macroscopic averaging procedures along the interface-normal direction. The corresponding mathematical definitions and alignment procedures are summarized in Table S1.

The planar-averaged potential provides the local variation of the electrostatic landscape across the heterostructure, whereas the macroscopic averaging removes short-wavelength oscillations associated with atomic layering and enables a physically meaningful description of long-range potential variations across the interface region.

Layer-Resolved Charge Redistribution

The spatial redistribution of electronic charge induced by interface formation was quantified using a layer-resolved Bader charge analysis. Atomic charges were grouped into slab-resolved layers parallel to the interface plane and referenced to bulk-like LATP layers sufficiently far from the interface in order to isolate the interfacial contribution. The mathematical definitions of the layer-resolved charge and relative charge-transfer descriptors are provided in Table S1.

These layer-resolved profiles provide a direct measure of the spatial extent and localization of interfacial charge redistribution across the LATP/LiF interface.

Table S1: Summary of the electrostatic descriptor framework used to quantify charge redistribution, potential alignment, dipole formation, and electric-field response across LiF/LATP interfaces. For each descriptor, the mathematical definition, input quantities, spatial averaging procedure, interface-region definition, and normalization/alignment scheme are specified.

**Descriptor**	**Mathematical definition**	**Input data**	**Averaging / alignment procedure**
Planar electrostatic potential	$V_{\text{planar}}(z) = \frac{1}{A} \iint V(x, y, z)\, dx\, dy$	LOCPOT	Planar averaging parallel to the interface; referenced to vacuum plateau
Macroscopic electrostatic potential	$V_{\text{macro}}(z) = \frac{1}{\Delta z} \int_{z-\Delta z/2}^{z+\Delta z/2} V_{\text{planar}}(z')\, dz'$	Planar LOCPOT	Sliding-window averaging along interface normal to remove atomic-scale oscillations
Charge-density difference (CDD)	$\Delta\rho(\mathbf{r}) = \rho_{\text{LiF/LATP}}(\mathbf{r}) - \rho_{\text{LATP}}(\mathbf{r}) - \rho_{\text{LiF}}(\mathbf{r})$	CHGCAR	Computed using identical supercell geometries for interface and isolated slab references
Planar-averaged CDD	$\overline{\Delta\rho}(z) = \dfrac{1}{A} \iint_A \Delta\rho(x, y, z)\, dx\, dy$	CDD grid	Planar averaging over the interface-parallel directions across the full slab thickness
Interface dipole moment	$\mu_z = \displaystyle\int (z - z_0)\, \Delta\rho(z)\, dz$	Planar-averaged CDD	Dipole moment of the planar-averaged CDD profile along the interface-normal direction, using z_0 as the chosen interface reference plane
Layer-resolved charge redistribution	$Q_i = \sum_{j \in i} q_j$	Bader charges	Atomic charges summed within slab-resolved interface layers adjacent to the interface plane
Electric-field distribution	$E_z(x, z) = -\partial \langle V(x, z) \rangle_y / \partial z$	LOCPOT	Derived from the gradient of the y-averaged electrostatic potential within $\sim$ 25 Å of the interface

Charge-Density Difference and Interface Dipole Moment

Electronic redistribution associated with interface formation was further characterized using charge-density difference (CDD) analysis constructed from self-consistent interface and isolated slab charge densities calculated within identical supercell geometries. Planar averaging of the charge-density difference over the interface-parallel plane yields the planar-averaged CDD profile, $\overline{\Delta\rho}(z)$, along the interface-normal direction. The first moment of this planar-averaged profile was then used to evaluate the interface dipole moment and its spatial localization. The mathematical definitions of the charge-density difference, planar-averaged CDD profile, and interface dipole moment are summarized in Table S1.

Interfacial Electric-Field Distribution

To visualize the electrostatic response across the interface, two-dimensional maps of the out-of-plane electric field were constructed from the electrostatic potential obtained from the `LOCPOT` output. For the two-dimensional representation, the electrostatic potential was first averaged only along the in-plane y direction, while retaining the spatial dependence along x and z:

$$\langle V \rangle_y(x, z) = \frac{1}{L_y} \int_0^{L_y} V(x, y, z)\, dy, \tag{1}$$

where L_y is the cell length along the y direction. The corresponding local out-of-plane electric field was then evaluated as

$$E_z(x, z) = -\frac{\partial \langle V \rangle_y(x, z)}{\partial z}. \tag{2}$$

In addition, the one-dimensional planar-averaged electrostatic potential was defined by averaging over the full interfacial plane,

$$\overline{V}(z) = \frac{1}{L_x L_y} \int_0^{L_x} \int_0^{L_y} V(x, y, z)\, dx\, dy, \tag{3}$$

where L_x and L_y are the in-plane cell dimensions. The corresponding one-dimensional electric-field profile normal to the interface is given by

$$E_z(z) = -\frac{d\overline{V}(z)}{dz}. \tag{4}$$

Two-dimensional maps of $E_z(x, z)$ were constructed within a region extending approximately 25 Å from the interface in order to resolve the spatial structure of the interfacial electrostatic response.

Ti-3d PDOS from Representative DFT+U Calculations

To further assess the robustness of the interfacial electronic-structure interpretation beyond the PBE level, a representative spin-polarized DFT+U calculation was performed for the relaxed LiF(100)/LATP(012) heterostructure. The on-site Hubbard correction was applied to Ti-3d states using the Dudarev formalism, with $U_{\text{eff}} = U - J = 3.5$ eV. This calculation is intended as a representative robustness check, rather than a complete DFT+U survey over all LiF crystallographic orientations. The geometry optimization within the DFT+U framework converged successfully and yielded a final total energy of TOTEN = −2574.963 eV.

A subsequent static DFT+U calculation was then carried out on the fully relaxed structure to obtain a well-converged electronic ground state prior to density-of-states analysis. The total magnetization remained zero throughout the calculation, confirming that the system does not evolve toward a spin-polarized magnetic state within the DFT+U description.

Based on the converged static electronic structure, the Ti-3d PDOS was analyzed and is shown in Figure S1. The energy scale is referenced to the Fermi level ($E_F = 0$ eV), indicated by the vertical dashed line. The spin-resolved Ti-3d components exhibit nearly perfect symmetry between spin-up and spin-down channels, consistent with the absence of

local magnetic moments at the Ti sites.

Importantly, no pronounced occupied Ti-3d states are observed at or immediately below the Fermi level. Instead, the dominant Ti-3d spectral weight remains located in the unoccupied region above E_F, indicating that the inclusion of on-site Hubbard corrections does not introduce clear Ti-centered reduction at the interface. These representative DFT+U results support the robustness of the electronic-structure interpretation for the tested LiF(100)/LATP(012) interface, while the orientation-dependent comparison remains based on the consistent PBE-level analysis across all three interfaces.

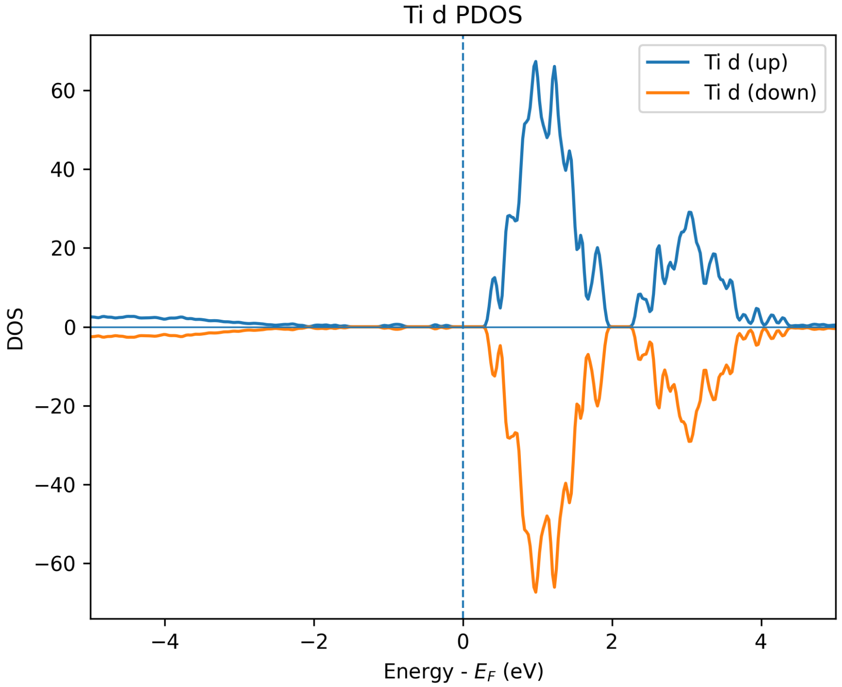

Figure S1: Representative spin-polarized DFT+U PDOS for the relaxed LiF(100)/LATP(012) heterostructure, obtained using $U_{\text{eff}} = U - J = 3.5$ eV for Ti-3d states. This calculation is provided as a robustness check for the electronic-structure interpretation of the LiF(100)/LATP(012) interface and should not be interpreted as a complete DFT+U survey over all LiF orientations.

Zoomed-in PDOS: Identification of Orientation-Dependent Interfacial States

To further clarify the presence or absence of interface-induced electronic states, a magnified view of the PDOS in the vicinity of E_F is provided in Figure S2. This zoomed-in representation enables a clearer comparison of orientation-dependent features that may contribute to electronic leakage pathways across the LiF/LATP interface. In particular, the appearance of a localized unoccupied interfacial state in selected orientations supports the interpretation of orientation-sensitive formation of interface-induced localized electronic states at the interface, consistent with the PDOS and CDD analyses discussed in the main text.

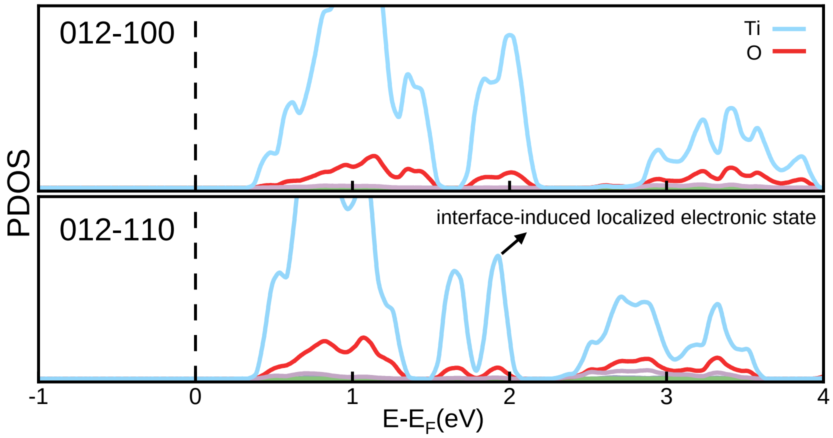

Figure S2: Zoomed-in PDOS for the LiF/LATP interface, highlighting orientation-dependent interfacial states relevant to possible electronic leakage pathways.

Bader Charge Analysis of Ti Ions

This section presents Bader charge data for Ti ions in the LiF/LATP heterostructures and LATP reference models. Table S2 summarizes the Ti Bader charge values for different LiF crystallographic orientations under neutral and negatively charged conditions. Table S3 compares the Ti Bader charges obtained for bulk and slab models of LATP.

Table S2: Bader charge transfer of Ti ions in LiF/LATP heterostructures with different LiF crystallographic orientations under neutral and negatively charged conditions. All values are given in units of $|e|$ and rounded to two decimal places.

Ti atom	012–100		012–110		012–111	
	Neutral	Charged	Neutral	Charged	Neutral	Charged
Ti-1	1.96	1.97	1.96	1.97	1.97	1.97
Ti-2	2.03	2.05	2.03	2.05	2.04	2.06
Ti-3	1.97	1.98	1.97	1.98	1.96	1.97
Ti-4	1.97	1.97	1.96	1.97	1.96	1.96
Ti-5	1.96	1.97	1.96	1.97	1.96	1.97
Ti-6	1.96	1.97	1.95	1.96	1.95	1.96
Ti-7	1.97	1.97	1.96	1.96	1.96	1.96
Ti-8	2.01	2.03	2.01	2.03	1.95	1.95
Ti-9	1.97	1.97	1.96	1.96	1.96	1.96
Ti-10	1.99	2.02	1.99	2.02	2.00	2.02
Ti-11	1.98	1.99	1.99	2.00	2.00	2.01
Ti-12	2.00	2.02	1.99	2.00	1.97	1.97
Ti-13	1.99	2.00	1.94	1.95	1.93	1.94
Ti-14	1.97	1.98	1.97	1.97	2.00	2.01
Ti-15	1.97	1.97	1.96	1.96	1.98	1.98
Ti-16	2.00	2.02	2.00	2.02	2.01	2.03
Ti-17	2.00	2.01	2.00	2.01	2.01	2.02
Ti-18	2.03	2.04	2.03	2.04	2.04	2.05
Ti-19	1.97	1.97	1.96	1.96	1.95	1.95
Ti-20	1.99	1.99	1.98	1.98	1.95	1.95

Table S3: Comparison of Ti Bader charge transfer in bulk and slab models of LATP. All values are reported in units of $|e|$. A dash indicates Ti atoms not present in the bulk reference cell.

Ti atom	Bulk	Slab
Ti-1	1.963	1.971
Ti-2	1.952	1.967
Ti-3	1.967	1.968
Ti-4	1.957	1.958
Ti-5	1.956	1.963
Ti-6	1.956	1.967
Ti-7	1.958	1.966
Ti-8	1.962	1.960
Ti-9	1.955	1.961
Ti-10	1.961	2.031
Ti-11	–	1.987
Ti-12	–	2.028
Ti-13	–	2.001
Ti-14	–	2.001
Ti-15	–	1.975
Ti-16	–	1.995
Ti-17	–	1.997
Ti-18	–	2.010
Ti-19	–	1.988
Ti-20	–	1.959
